# Selective Laser Trabeculoplasty Protects Glaucoma Progression in the Initial Primary Open-Angle Glaucoma and Angle-Closure Glaucoma after Laser Peripheral Iridotomy in the Long Term

**DOI:** 10.1155/2019/4519412

**Published:** 2019-12-21

**Authors:** Natalia Ivanovna Kurysheva, Lyudmila Vyacheslavovna Lepeshkina

**Affiliations:** ^1^Head of the Consultative and Diagnostic Department of the Ophthalmological Center of the Federal Medical and Biological Agency of the Russian Federation, the A.I. Burnazyan Federal Medical and Biophysical Center of FMBA, Ophthalmological Department of the Institute of Improvement of Professional Skill of FMBA, 15 Gamalei Street, Moscow 123098, Russia; ^2^The Ophthalmological Center of the Federal Medical and Biological Agency of the Russian Federation, 15 Gamalei Street, Moscow 123098, Russia

## Abstract

**Purpose:**

To compare the ability of SLT in preventing glaucoma progression in the initial primary angle-closure glaucoma (PACG) after laser peripheral iridotomy and primary open-angle glaucoma (POAG) in the long term.

**Methods:**

60 patients with the initial stage of PACG after laser peripheral iridotomy and 64 initial POAG patients were recruited in a prospective study. Complete success of selective laser trabeculoplasty (SLT) was defined as a 20% intraocular pressure (IOP) reduction with topical hypotensive medications without any hypotensive intervention. Pre-SLT rate of progression and post-SLT rate of progression (ROP) was detected in the both groups by means of the trend and the event analysis of perimetry, the Guided Progression Analysis, and the optical coherence tomography- (OCT-) based negative trend for either the thickness of the peripapillary retinal nerve fiber layer (RNFL) or ganglion cell complex (GCC).

**Results:**

IOP decreased significantly after SLT in both the groups. 30% in PACG and 19% in POAG had the progression according to perimetry and 49% in PACG and 40% in POAG had the progression, respectively, according to OCT. After SLT, ROP was reduced from −0.14 ± 0.39 dB/year to −0.08 ± 0.48 dB/year, *p*=0.034, in PACG and from −0.09 ± 0.36 dB/year to −0.04 ± 0.43 dB/year, *p*=0.021, in POAG. According to RNFL trend analysis, ROP was reduced from −1.86 ± 2.9 *μ*m/year to −1.38 ± 2.2 *μ*m/year, *p*=0.039, and from −1.24 ± 2.23 *μ*m/year to −0.76 ± 1.73 *μ*m/year, *p*=0.037, in PACG and POAG, and according to GCC, ROP was reduced from −1.88 ± 2.9 *μ*m/year to −1.34 ± 2.0 *μ*m/year, *p*=0.040, and from −1.35 ± 2.16 *μ*m/year to −0.91 ± 1.86 *μ*m/year, *p*=0.040, in PACG and POAG, respectively. ROP was significantly faster in PACD than in POAG between 2 and 6 years after SLT: −0.15 ± 0.46 dB/year and 0.02 ± 0.38 dB/year (*p*=0.042). However, it did not differ significantly according to OCT.

**Conclusion:**

SLT is an effective treatment for initial PACG after LPI and POAG that can prevent functional and structural deterioration in the long term.

## 1. Introduction

Primary angle-closure glaucoma (PACG) is one of the most severe forms of glaucoma. 16 million people suffer from PACG worldwide [1]. Moreover, it progresses faster than primary open-angle glaucoma (POAG) [2] with a rapid progression in more than 57% of patients [3]. A quarter of all patients has one blind eye, and 4 million patients with PACG are totally blind [4]. The current mostly used treatment for this form of glaucoma is laser peripheral iridotomy (LPI) [5, 6]. However, most patients after the procedure are required to use topical hypotensive eye drops [7]. Another method of treating PACG is phacoemulsification or lens extraction, which also requires the use of hypotensive eye drops [8]. An alternative treatment method of PACG with uncompensated intraocular pressure (IOP) after LPI is selective laser trabeculoplasty (SLT). There have been only a few publications in the literature demonstrating efficacy of this treatment method in PACG [9–11]. Currently, SLT is the most common method of laser treatment of primary open-angle glaucoma (POAG) [12, 13]. Recently, we have demonstrated the efficacy of SLT for patients with PACG following a YAG laser peripheral iridotomy (PI) and studied the predictors of its outcome [14]. We revealed that “the one-year efficacy of SLT in POAG and PACG after LPI was high, but it was reduced in the long-term period”. We supposed that it could lead to increase in glaucoma progression. It is an interesting fact that the comparative efficacy of SLT in protecting from PACG and POAG progression in the long-term period has not been studied before.

The purpose of this study is to assess the ability of SLT in preventing glaucoma progression in PACG in the long-term and to compare it with the primary POAG.

## 2. Patients and Methods

### 2.1. Study Design and Patients

This was a longitudinal prospective study. It was approved by the Institutional Review Board of the Federal Medical and Biological Agency of the Russian Federation and was conducted in accordance with the provisions of the Declaration of Helsinki.

The participants were recruited using an electronic medical database of A.I. Burnazyan Federal Medical and Biophysical Center of FMBA Eye Center between April 2010 and May 2012. The patients who met the criteria described below were consecutively included by retrospective medical record review.

The recruited patients were treated with LPI and SLT in the PACG group and only SLT in the POAG group. After the treatment, all the patients were examined in one month and every four months annually within the period of up to 6 years. In accordance with our Institutional Review Board, the written informed consent was obtained from all participants before the intervention.

All the examinations were carried out during every visiting at 10.00–12.00 AM. The patients, who had no less than six tests of perimetry and OCT before LPI/SLT and fulfilled the inclusion criteria, were included in this study.

POAG was diagnosed on the basis of an open anterior chamber angle (no less than 30°, see the study examinations below) and typical changes in the optic disc, detected during ophthalmoscopy (abnormal proportions of neural rim, glaucomatous excavation of optic disc, peripapillary atrophy, retinal nerve fiber layer wedge-shaped defects close to the edge of the optic disc, and hemorrhage at the optic disc edges). The diagnosis of glaucoma according to ophthalmoscopy was confirmed by two independent glaucoma specialists. The results of standard automated perimetry (SAP) performed at Humphrey perimeter (Carl-Zeiss Meditec, Dublin, CA) were abnormal. Glaucomatous visual field defects were determined as having a cluster of 3 or more nonedge points with *p* < 0.05 and at least 1 point with *p* < 0.01 in the pattern deviation probability plot; pattern standard deviation (PSD) of less than 5%; or glaucoma hemifield test results outside normal limits. The initial stage of glaucoma was based on the detection of average MD measurements over the entire follow-up using the Hodapp–Parrish–Anderson grading scale, and early glaucoma was defined as MD > −6 db [15].

Primary angle-closure glaucoma was defined as the presence of angle-closure (defined as eyes in which at least 180 of the posterior pigmented trabecular meshwork were not visible on gonioscopy in the primary position of gaze with no indentation with glaucomatous optic neuropathy (defined as loss of neuroretinal rim with a vertical cup-to-disc ratio (VCDR) of ≥0.7 or between-eye VCDR asymmetry of >0.2, focal notching of the neuroretinal rim with a VF defect suggestive of glaucoma, or a combination thereof [16]).

### 2.2. Intervention

At first, the patients with PACG were exposed to LPI. SLT was performed not earlier than two months following the LPI, provided that the anterior chamber angle was not less than 180° (defined as visible pigmented TM for ≥180° on gonioscopy in the primary gaze) and that there was no goniosynechia, which was confirmed by gonioscopy of the anterior chamber angle.

The patients who had an active anterior segment inflammation, synechial closure of the anterior chamber angle and secondary causes of angle-closure such as subluxed lens, uveitis, trauma, and neovascular glaucoma, and those in whom corneal pathology obscured gonioscopic view to the angle, as well as patients who had underwent phacoemulsification of cataract or glaucoma filtration surgery, previous laser trabeculoplasty, refractive surgery, or iridoplasty were excluded. Exclusion criteria included presence of any media opacities that prevented good quality OCT scans, or any retinal (including posterior vitreous detachment, retinal vein occlusion, and diabetic retinopathy) or neurological disease other than glaucoma, which could confound the evaluations.

Patients were also excluded if the fellow eye was blind. If both eyes were eligible, they were both treated by SLT, but only the right eye was included in the analysis.

The preoperative examination included autorefractometry, visometry, gonioscopy, optical coherence tomography (OCT) (OCT RTVue-100, Optovue, Inc., Fremont, CA), OCT of anterior ocular segment with measuring the anterior chamber angle (Optovue Rtvue 100 (CA), pachymetry (SP-100 Tomey, Germany), biometry (Lenstar LS 900, Haag-Streit Diagnostics, Switzerland), perimetry (Humphrey, Carl-Zeiss Meditec, Dublin, CA), SITA Standard 24-2, biomicroscopy, and Goldman tonometry.

The indication for SLT and for repeated SLT in the both groups were as follows: (1) a high level of IOP if for its reduction more than one hypotensive eye drop a day was required or (2) if there were some contraindications or intolerance of the eye drops or (3) if the progression of glaucoma had been detected despite a normal IOP level.

Before the repeated SLT, the configuration of the anterior angle was examined carefully. Special attention was paid to the formation of synechial closure. The contraindications for the repeated SLT were the same as for the first intervention.

SLT was performed under topical anesthesia using the Latina lens (Ocular Instruments Inc., WA, USA). Power was initially set at 0.6 mJ and increased in 0.1mJ steps until small bubbles appeared from the treated area of the TM. Contiguous nonoverlapping 50–80 laser applications were performed over 180–360° using Lasersx Solo ND:YAG laser (Ellex Medical Lasers Limited, Adelaide, Australia). In PACG eyes, nonoverlapping shots were placed onto at least 180° of the visible TM, avoiding areas of angular synechiae. The pulse energy level ranged from 0.6 to 1.0 mJ depending on the degree of TM pigmentation.

All eyes were pretreated with brimonidine tartrate, 0.15%, and pilocarpine hydrochloride 2.0% (if it was necessary in PACG eyes) prior to the procedure. 0.5% proximetacaine solution was used as topical anesthesia. Inflammatory reaction after SLT was assessed in points: no inflammation: 0, reduction of the pupillary light reflex: 1, reduction of the pupillary light reflex and iris hyperemia: 2, and precipitates and Tyndall phenomenon in the anterior chamber: 3. To prevent the possible inflammation, all patients immediately after SLT, and also in some cases (according to the indications), were prescribed topical nonsteroidal anti-inflammatory (NSAI) drug, indomethacin (indocollyre) 0.1% (Chauvin Inc., UK), on the first day after SLT and for the first three days. No steroids were administered for the operated eyes. Dorzolamide hydrochloride 2% was applied twice daily on the operation day to prevent reactive hypertension in all eyes.

Complete hypotensive success of SLT was defined as a 20% IOP reduction with topical hypotensive medications without any hypotensive intervention (repeated SLT, antiglaucoma surgery, and phacoemulsification of cataracts).

### 2.3. Detection of Glaucoma Progression

The Guided Progression Analysis (GPA) software on the Humphrey Field Analyzer II was used to detect glaucoma progression. The analysis includes a VF trend analysis defined with either VFI or MD and a pointwise event analysis. The event analysis defined progression as a significant change (compared to two significant VFI or MD, negative slope was observed [17]). The probability levels were considered to be statistically significant where *p* was less than 0.05 for the slope of the global 24-2 area. Only significant values were selected for the calculation of mean progression rates. SAP was performed every 6 months. From the entire dataset containing visual fields obtained with the Humphrey Field Analyzer, those patients with ≥8 visual field examinations were included [17]. The VF progression endpoint was detected when either the event analysis or the trend analysis showed significant progression. Only visits with both VF and OCT data were used.

The peripapillary nerve fiber layer (NFL) and macular ganglion cell complex (GCC) were imaged and measured by FD-OCT (RTVue, Optovue, Inc., Fremont, CA, USA). During each visit, participants had three GCC and optic nerve head (ONH) scans. Only ONH scans with a signal strength index (SSI) above 37 and GCC scans above 42 were selected for analysis. Measurements of qualified scans in the same visit were averaged. The macular GCC scan covered a 7 by 7 mm square area in the macula. Scans were centered 0.75 mm temporal to the fovea to improve the coverage of the temporal macula. The macular GCC thickness was defined as the combination of NFL, GCL, and inner plexiform layer. The automated Optovue software derived a 6 mm diameter GCC thickness map centered 0.75 mm temporal to fovea. The ONH concentric (1.3–4.9 mm diameter) scans were centered on the optic disc and automatically registered with the 3D disc scan to provide the disc margin information. The NFL thickness profile at *D* = 3.4 mm was resampled on the NFL map recentered according to the detected optic disc center. The RTVue software (version 6.12) was used to provide the following OCT image-derived measurements: the overall GCC thickness map and the overall NFL thickness profiles. We used two OCT parameters to track glaucoma disease status: overall GCC thickness and NFL thickness. At each visit, the series of OCT thickness parameters from baseline to the current visit was fit over time. Progression was defined at the visit where a significant (*p* < 0.05) negative slope (thinning trend) was observed. The visit at which significant progression trend was first observed was recorded as the date of progression detection [18].

To eliminate the interference of cataract on the visual field measurements, we also excluded eyes that experienced significant cataract progression any time during the follow-up. A significant cataract progression is defined as confirmed worsening of visual acuity scores by two or more lines at two or more follow-up visits and confirmed clinical cataract progression assessment at two or more follow-up visits.

### 2.4. Main Outcome Measures

(i) *Primary outcome*. Visual field and structural progression that were expressed in the rate of VF loss, the rate of average RNFL thinning, and the rate of average GCC thinning.

(ii) *The second outcome measures*. 20% IOP reduction after SLT with topical hypotensive medication.

### 2.5. Statistical Analysis

The obtained results were statistically processed with the Statistical Package for Social Sciences (SPSS) 16.0 for Windows software using the variational statistics method. The mean and proportions were compared using Student's *t*-test and the *X*^2^ test. The Mann–Whitney *U* test was used to find differences in progression of glaucoma per year between patients' groups, amount of topical hypotensive eye drops, and other continuous variables. The values of MD, RNFL, and GCC were compared using the Wilcoxon test. Furthermore, covariance analysis for baseline IOP was used to compare the mean IOP change between the groups. The Kaplan–Meier survival analysis was used to assess the likelihood of continued IOP control and to compare time-to event data for evaluation of MD, RNFL, and GCC change for 6 years after SLT in each group. The log rank test was applied for distinguishing the PACG and POAG groups. The critical level of statistical significance was *p* < 0.05.

## 3. Results

The present analysis included 62 out of 120 total initial PACG eyes and 64 out of 120 total initial POAG eyes after applying the minimum complete follow-up visit requirements and after excluding 17 PACG eyes and 11 POAG eyes showing significant cataract progression. The follow-up period amounted to 40.67 ± 1.43 months for PACG eyes before SLT and 39.49 ± 20.48 after SLT and 37.29 ± 19.13 months before SLT and 42.68 ± 21.18 after SLT for POAG eyes. If both eyes were eligible, they were both treated by SLT but only the right eye was included into the analysis.

According to Table 1, at baseline, the groups of patients were homogeneous, except for anterior-posterior axis (*p*=0.001), the anterior chamber depth (*p*=0.001), and the size of the anterior chamber angle in the upper half (*p*=0.003).The mean (SD) extent of angle treated by SLT as well as the mean (SD) energy did not differ significantly in POAG and PACG patients (Table 1).

The frequency of mild and moderate inflammatory reaction was approximately the same during the first day after SLT in both groups. No serious inflammatory reaction was observed in either case. Mild inflammation was obtained in 5.88% of POAG eyes and in 7.69% PACG eyes (*p*=0.72) and moderate inflammation in 4.41% POAG eyes and in 6.15% PACG eyes (*p*=0.7).

During the entire follow-up period, there was a significant IOP decrease in comparison with the baseline IOP in both POAG and PACG eyes, while the groups did not differ significantly (Table 1).

**Figure 1:**
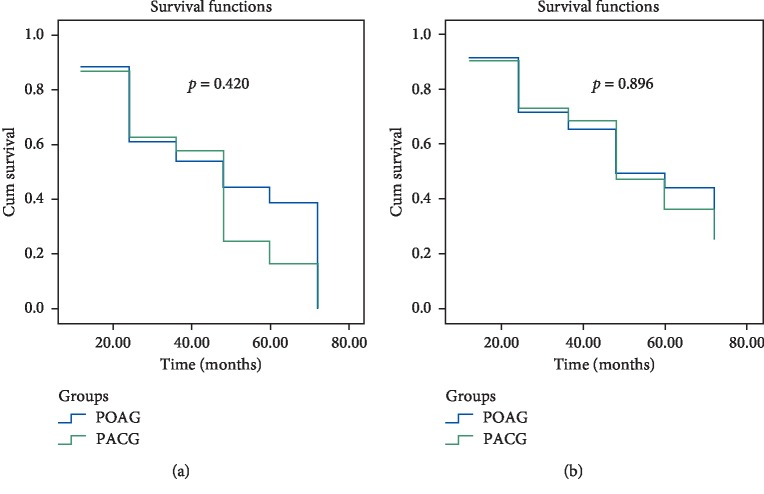
Kaplan–Meier survival curves for POAG and PACG according to criteria of success as a 20% IOP reduction with topical hypotensive medications. (a) Without repeated SLT. (b) With repeated SLT.

**Figure 2:**
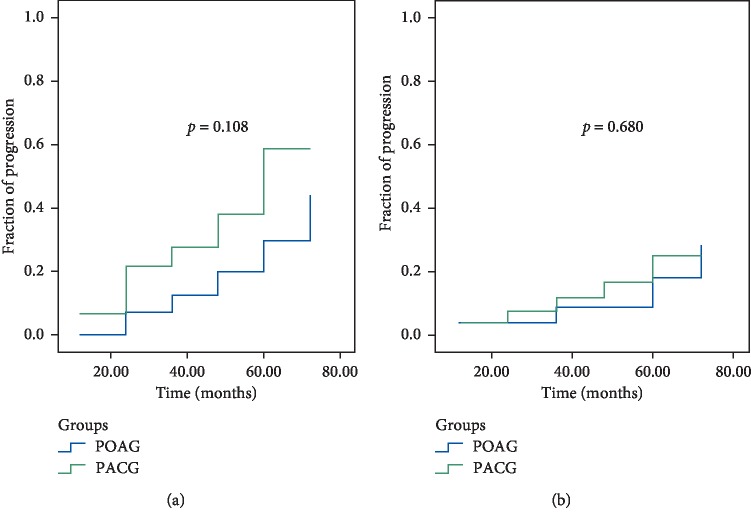
Kaplan–Meier plots of glaucoma progression detected by visual field among PACG and POAG patients after SLT. (a) Without repeated SLT. (b) With repeated SLT.

**Figure 3:**
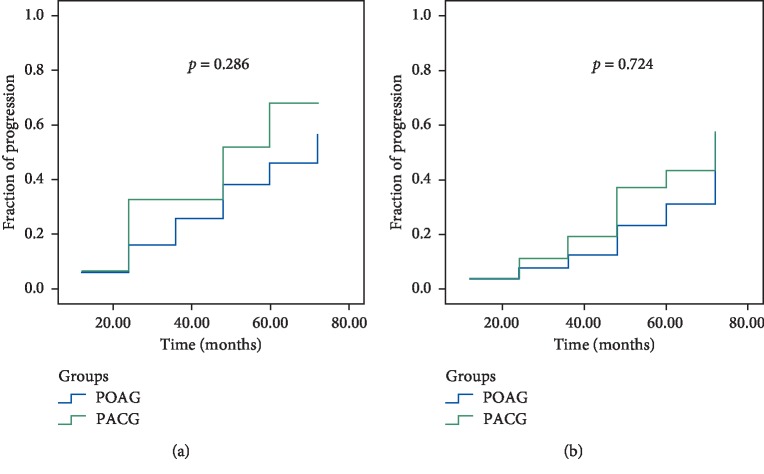
Kaplan–Meier plots of glaucoma progression detected by OCT (RNFL) among PACG and POAG patients after SLT. (a) Without repeated SLT. (b) With repeated SLT.

**Figure 4:**
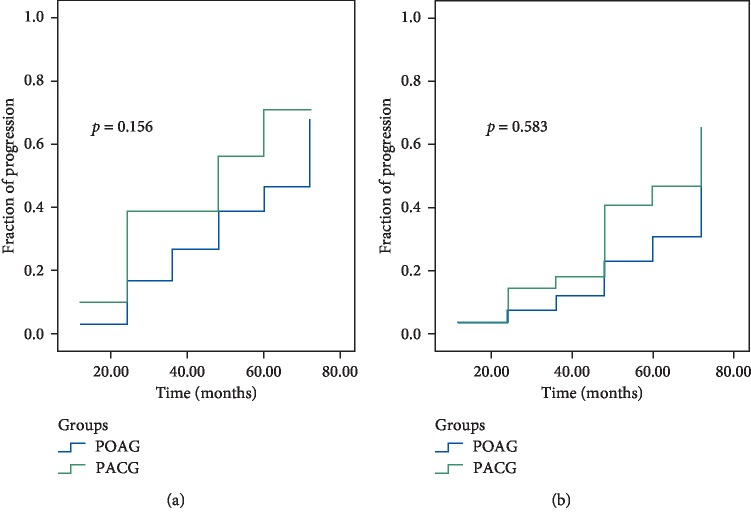
Kaplan–Meier plots of glaucoma progression detected by OCT (GCC) among PACG and POAG patients after SLT. (a) Without repeated SLT. (b) With repeated SLT.

Figure 1 shows the results of the SLT efficacy assessment, which demonstrates the overall probability of its success in POAG and PACG in the follow-up period. Complete success was achieved in 86% and 88% eyes in PACG and POAG, respectively, in 1 year and in 2% and 15% eyes in 6 years. After repeated SLT, the complete success exceeded 89% and 90% in one year and 34% and 36% in 6 years, respectively, in PACG and POAG. Moreover, glaucoma progression was reduced significantly in the eyes with repeated SLT in both glaucoma groups (Figures 2(b), 3(b), and 4(b)).

The rate of progression (ROP) was significantly faster in PACD than in POAG between 2 and 6 years: according to the GPA Humphrey test, −0.15 ± 0.46 dB/year and 0.02 ± 0.38 dB/year (*p*=0.042), respectively (Figure 2). According to OCT trend analysis of RNFL, the difference was not significant, i.e., −1.56 ± 1.35 *μ*m/year in PACG and −0.73 ± 1.39 *μ*m/year in POAG (*p*=0.068) (Figure 3) and according to OCT trend analysis of GCC, the difference was not significant, i.e., −1.64 ± 2.32 *μ*m/year in PACG and −0.82 ± 1.46 *μ*m/year in POAG (*p*=0.043) (Figure 4). There was no significant difference in glaucoma progression between PACG and POAG in the other periods of examination.

**Figure 5:**
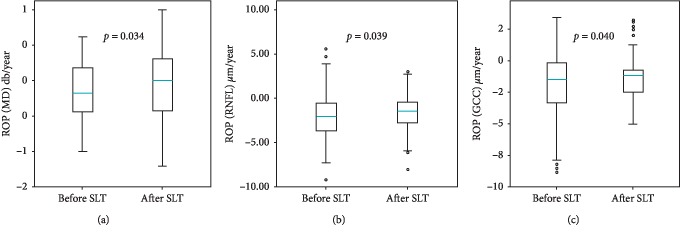
Rate of progression detected by means of Humphrey GPA (a), RNFL (b), and GCC (c) change before (the left plots on the diagrams) and after (the right plots) SLT in PACG.

**Figure 6:**
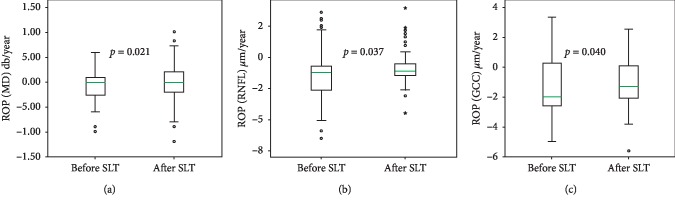
Rate of progression detected by means of Humphrey GPA (a), RNFL (b), and GCC (c) change before (the left plots on the diagrams) and after (the right plots) SLT in POAG.

We have revealed a significant decrease of ROP in both studied groups after SLT compared to ROP within the period preceding SLT: in PACG, according to the GPA Humphrey test, it was reduced from −0.14 ± 0.39 dB/year to −0.08 ± 0.48 dB/year, *p*=0.034 (Figure 5(a)); according to RNFL trend analysis, it was reduced from −1.86 ± 2.9 *μ*m/year to −1.38 ± 2.2 *μ*m/year, *p*=0.039 (Figure 5(b)); and according to GCC trend analysis, it was reduced from −1.88 ± 2.9 *μ*m/year to −1.34 ± 2.0 *μ*m/year, *p*=0.040 (Figure 5(c)).  Figure 6 demonstrates a significant reduction of ROP after SLT in the POAG group from −0.09 ± 0.36 dB/year to −0.04 ± 0.43 dB/year, *p*=0.021 (Figure 6(a)), from −1.24 ± 2.23 *μ*m/year to −0.76 ± 1.73 *μ*m/year, *p*=0.037 (Figure 6(b)), and from −1.35 ± 2.16 *μ*m/year to −0.91 ± 1.86 *μ*m/year, *p*=0.040 (Figure 6(c)) according to MD, RNFL, and GCC analysis, respectively.

## 4. Discussion

Though the efficacy and safety of SLT have been shown in different studies both in POAG [12, 13] and in PACG [9–11], it is not clear if SLT can affect glaucoma progression. Meanwhile, an evaluation of the rate of disease progression and detecting the risk of visual impairment and blindness are an important aspect of glaucoma management.

To the best of our knowledge, this is the first study demonstrating that SLT leads to a reduction of glaucoma progression both in PACG and POAG. There has been only one publication devoted to the comparison of the ROP in POAG and PACG. Thus, a 5-year retrospective study by Lee et al. has revealed a more rapid progression in PACG patients. Moreover, the highest IOP detected in each year throughout the study was the only progression factor in PACG patients [2]. Following up PACG patients for over 10 years, Verma et al. noted the progression in 15.8% patients, with the average ROP of −0.12 dB/year [3]. These data differ from those obtained in our study. Thus, according to the GPA-analysis for a 6-year follow-up, the ROP amounted to 23% in POAG and 21.8% in PACG. The data discrepancy between the study by Verma and our study may be explained by various methods for estimating progression by means of perimetry: pointwise linear regression analysis (PLR, Progressor software) applied in the Verma study and the Guided Progression Analysis (GPA) applied in the present study. The progression criteria in this software are different.

Using the same software (GPA) and also observing patients for 6 years, Zhang and co-authors have revealed the progression of POAG in 18.7% cases [19]. The same authors have also found out that the glaucoma progression is detected twice often using the OCT method (38.9%, *p* < 0.001), especially when analyzing the GCC changes. These data coincide with ours, because overall progression in both the groups detected using the OCT method amounted to 36.8% (50 eyes) in comparison with SAP: 19.1% (26 eyes), *p*=0.001. According to the present study, the ROP in PACG was the same as that obtained by Verma [3] and was 2.5 times higher than that in POAG at the period of from 2 to 6 years after SLT. These data are similar to the results obtained by Lee [2].

The present study demonstrates the efficacy and safety of SLT in treating PACG after LIT. While SLT has been used for treating POAG for more than 10 years, it is a relatively new approach to the treatment of PACG. This is due to the previous opinion that the anterior chamber angle structures are not available for trabeculoplasty in PACG. In 2012, Shiota et al. carried out a comparative analysis of the anterior chamber angle structures in the patients with chronic PACG and POAG using the method of scanning electron biomicroscopy. The results showed similar histological changes in TM in both groups, which made it possible to consider the possibility of performing SLT in PACG after LPI with the same degree of safety as in POAG [20].

The most frequent adverse reactions of SLT in both glaucoma forms are mild irritation during the first days after the procedure, reactive IOP rise, corneal syndrome, bleeding, and transient changes in corneal endothelium [21, 22]. The literature describes the cases of such severe complications in the SLT postoperative period as haze and corneal edema [23]. In this study, all patients had no complications after SLT. The results of the present study demonstrate no significant complications in any glaucoma group. Moreover, the corneal endothelium that was affected in the nearest days after SLT has been restored by the end of the first month after SLT in the most of patients.

According to the literature, SLT in POAG can decrease IOP by 30% of the baseline IOP, which is comparable in its effect with the use of prostaglandin analogues (PGAs) [12, 13]. There have been only a few observations concerning PACG on this subject [9–11]. Thus, one of them compared the efficacy of SLT with the efficacy of treatment using PGAs [10]. Decrease in IOP relative to the baseline IOP was observed in 60% after SLT and in 84% after the treatment with PGAs. In 6 months after the procedure, IOP decreased by 4.0 mm Hg averagely in the SLT group compared to 4.2 mm Hg in the PGAs group (*p*=0.78), which amounted to 16.9% and 18.5%, respectively (*p*=0.52). The authors concluded that the hypotensive effect of SLT in PACG after the previously performed LPI is comparable with the effect of the treatment with PGAs.

The study by Ali Aljasim et al. contains a comparative analysis of safety and efficacy of SLT with the primary angle-closure (PAC)/PACG and POAG within the period from 2011 to 2013. The success rate of achieving a clinically significant decrease in IOP (by 20% or more from the baseline IOP) was 84.7% in the PAC/PACG group and 79.6% in the POAG group (*p*=0.47) [11]. According to Ho et al., SLT reduces IOP by 20% without increasing the topical hypotensive regimen during the first 6 months after the procedure in almost 50% patients with PAC [9].

In contrast to the studies described above, we were the first who observed the efficacy of SLT in PACG in a long-term period (6 years) and found that it was comparable with SLT in POAG. Moreover, when hypotensive drops had to be prescribed, the normalization of IOP in PACG was achieved by their smaller dosage in both glaucoma forms.

It has been emphasized in different studies that high IOP is the main risk factor of glaucoma progression [24–28]. According to the results of the present study, the efficacy of SLT was less in PACG during the period of 24–72 months (Figure 1), which may explain the reason for its higher progression compared to PAOG at the same period (Figures 2–4). Moreover, we consider this period to be the most important in glaucoma monitoring for the propria strategy of treatment or its change: the repeated SLT, lens/cataract extraction, etc. It is worth noting that eyes with repeated SLT demonstrated less progression compared with the eyes with only one SLT in both glaucoma groups. Moreover, it was revealed that SLT decreased the rate of structural deterioration, measured by GCC trend analysis, by 1.4 times in PACG and by 1.5 times in POAG, and functional deterioration by 1.75 and 2.2 times in PACG and POAG, respectively. This may be explained by a significant reduction of IOP after SLT.

The frequency of repeated SLT did not differ significantly between the studied groups (Table 1); however, it was higher than reported by Narayanaswamy and co-authors [10], who followed the patients during 6 months after the procedure. Probably, this is due to a longer follow-up period in our study.

Our study has several limitations that must be acknowledged. First, we observed the patients only with an initial stage of primary glaucoma. Moderate and advanced stages of PACG are characterized by more pronounced damage of TM; hence, these eyes may be more tolerant to SLT.

Second, our study is prospective as the patients were recruited by retrospective medical record review and the rate of visual field deterioration was assessed on the basis of the perimetry data before laser treatment. However, all the recruited patients had no less than six tests of perimetry before LPI/SLT that permitted to calculate the rate of glaucoma progression.

Third, we did not observe the PACG eyes, which had undergone phacoemulsification of cataract after SLT, as it was not the purpose of this study. We expect that these eyes could demonstrate the better results in regard to preserving structure and function in the long term. This is the task for future research. Previously, we revealed that the efficacy of SLT in PACG was decreased in eyes with a smaller anterior chamber and a larger lens, both factors that would limit the exposure of ACA structures and reduce the amount of TM area available for treatment [14]. One may assume that cataract extraction after SLT may improve the IOP control.

The fourth limitation is that we did not assess the change of the anterior chamber and its angle in dynamic after SLT and consider only the IOP level that required the change of the treatment strategy. However, we paid attention to the appearance of new goniosynechia especially in the PACG eyes to avoid them during the repeated SLT treatment. We assume that change of the configuration of the anterior angle in PACG patients in the follow-up might be a reason for the lower efficacy of SLT in PACG during the period of 24–72 months that may explain the reason for its higher progression compared to PAOG at the same period.

The fifth limitation is that while assessing the GCC and RNFL trend analysis, we did not adjust for normal age-related structural thinning and for the OCT signal strength index. However, this method of progression analysis was admitted in the literature [19].

Finally, we did not compare eyes given 360° SLT treatment with those given 180° treatment, while some authors have emphasized a more pronounced hypotensive effect of 360° SLT treatment [29]. In the present study, the average amount of laser spots did not differ significantly between the groups that permitted the comparison between the PACG and POAG eyes. Moreover, according to the recent literature, even the 90° SLT is as effective as 360° SLT in a long-term period [30].

The interpretation of the glaucoma progression is rather challenging as many different factors may affect a glaucoma course [31–33]. However, in this study, we compared the glaucoma progression before and after SLT in the same patients. While comparing the patients with POAG and PACG, we considered that these groups were equated by age, gender, and the eye drops they were treated with after SLT. Our study has such strong points as a long follow-up period and assessment of ROP in primary glaucoma treated with SLT.

In conclusion, the conducted study has revealed that SLT both in patients with the initial PACG (with an open anterior chamber angle after LIT) and POAG allows reducing IOP in an effective and safe way within a period up to 6 years and may also decrease a functional and structural deterioration.

## Figures and Tables

**Table 1:** Clinical characteristics of the studied groups.

Characteristics	POAG	PACG	*p*
Age, years	68 ± 5.33	67 ± 7.37	0.367
Existence of glaucoma before SLT, years	2.34 ± 1.64	2.67 ± 1.43	0.321
Follow-up period after SLT, years	6.22 ± 1.54	6.75 ± 1.83	0.485
Corneal thickness, *μ*m	548 ± 32.83	545 ± 36.25	0.245
Endothelial cell count (cells/mm^2^) prior to SLT	2451 ± 132	2531 ± 145	0.401
Endothelial cell count (cells/mm^2^) 1 month after SLT	2482 ± 143	2343 ± 163	0.041
	*p* ^*∗*^=0.113	*p* ^*∗*^=0.001	
Anterior-posterior axis, mm	23.02 ± 1.59	21.88 ± 0.69	0.001
Anterior chamber depth, mm	3.35 ± 0.37	2.46 ± 0.45	0.001
Lens thickness, mm	4.82 ± 0.34	4.75 ± 0.32	0.102
RNFL, *μ*m	96.45 ± 12.04	97.56 ± 12.34	0.530
GCC, *μ*m	89.37 ± 7.64	90.15 ± 9.26	0.564
No. оf OCT tests	9.90 ± 1.67	10.67 ± 1.76	0.425
No. of visual field tests	9.55 ± 2.71	10.65 ± 3.03	0.276
Initial IOP, mm Hg	21.43 ± 2.40	22.19 ± 4.22	0.282
Last IOP, mm Hg	18.80 ± 1.98	18.46 ± 3.30	0.628
	*p* ^*∗*^=0.001	*p* ^*∗*^=0.001	
Initial MD, dB	−2.0 ± 1.19	−1.87 ± 1.65	0.156
Initial PSD, dB	2.34 ± 2.25	2.35 ± 1.96	0.417
Degree of TM pigmentation	2.2 ± 0.65	2.44 ± 0.43	0.456
Dimensions of ACA upwards (°)	32.2 ± 5.18	12.89 ± 3.95	0.002
Dimensions of ACA downwards (°)	30.5 ± 5.0	24.356 ± 4.21	0.120
Hypotensive regimen before SLT (average amount of eye drops)	1.25 ± 0.57	1.19 ± 0.49	0.403
Last average amount of eye drops after SLT	0.67 ± 0.59	0.48 ± 0.58	0.171
	*p* ^*∗*^=0.001	*p* ^*∗*^=0.001	
Mean energy/applications used, mJ	0.89 ± 0.10	0.88 ± 0.19	0.642
Average number of spots per eye	56.4 ± 5.59	53.5 ± 5.59	0.110
Repeat SLT	37.5%	34.17%	0.401
Average time for receiving the repeated SLT	16.3 ± 3.29	14.3 ± 4.31	0.322

Values are mean ± SD, unless otherwise noted. ACA, anterior chamber angle; GCC, ganglion cell complex; IOP, intraocular pressure; MD, mean deviation; PACG, primary angle-closure glaucoma; POAG, primary open-angle glaucoma; PSD, pattern SD; RNFL, retinal nerve fiber layer; TM, trabecular meshwork; SLT, selective laser trabeculoplasty. *p* indicates reliability of difference between the groups according to the Mann–Whitney *U* test. *p*^*∗*^ is calculated using the Wilcoxon signed-rank test.

## Data Availability

The data used to support the findings of this study have not been made available because we currently continue our research with the same group of patients using new technology swept-source OCT.
